# Exploring Pharmacological Mechanisms of Lavender (*Lavandula angustifolia*) Essential Oil on Central Nervous System Targets

**DOI:** 10.3389/fphar.2017.00280

**Published:** 2017-05-19

**Authors:** Víctor López, Birgitte Nielsen, Maite Solas, Maria J. Ramírez, Anna K. Jäger

**Affiliations:** ^1^Department of Pharmacy, Faculty of Health Sciences, Universidad San JorgeZaragoza, Spain; ^2^Department of Drug Design and Pharmacology, Faculty of Health and Medical Sciences, University of CopenhagenCopenhagen, Denmark; ^3^Department of Pharmacology and Toxicology, School of Pharmacy, University of NavarraPamplona, Spain

**Keywords:** essential oils, central nervous system, lavender, *Lavandula angustifolia*, *Lavandula officinalis*, SH-SY5Y cells, glutamate receptor

## Abstract

Lavender essential oil is traditionally used and approved by the European Medicines Agency (EMA) as herbal medicine to relieve stress and anxiety. Some animal and clinical studies reveal positive results in models of anxiety and depression although very little research has been done on molecular mechanisms. Our work consisted of evaluating the effects of lavender (*Lavandula angustifolia*) essential oil on central nervous system well-established targets, such as MAO-A, SERT, GABA_A_and NMDA receptors as well as *in vitro* models of neurotoxicity. The results showed that lavender essential oil and its main components exert affinity for the glutamate NMDA-receptor in a dose-dependent manner with an IC_50_ value of 0.04 μl/mL for lavender oil. In addition, lavender and linalool were also able to bind the serotonin transporter (SERT) whereas they did not show affinity for GABA_A_-benzodiazepine receptor. In three different models of neurotoxicity, lavender did not enhance the neurotoxic insult and improved viability of SH-SY5Y cells treated with hydrogen peroxide. According to our data, the anxiolytic and antidepressant-like effects attributed to lavender may be due to an antagonism on the NMDA-receptor and inhibition of SERT. This study suggests that lavender essential oil may exert pharmacological properties via modulating the NMDA receptor, the SERT as well as neurotoxicity induced by hydrogen peroxide.

## Introduction

Essential oils have a long tradition in pharmaceutical sciences as natural products with pharmacological, cosmetic, agrochemical and nutritional applications (Bakkali et al., 2008). The use of EO in form of aromatherapy or phytotherapy is widely extended, some of them being used as agents to relieve anxiety and stress (Setzer, 2009). Phytotherapy consists of the use of medicinal plants in order to prevent, cure or threat illnesses. Aromatherapy can be understood as a subdivision of phytotherapy and defined as the use of essential oils regarding therapeutic effects. These products have been used for centuries and are accepted in traditional or modern healthcare systems of medicine. Medicinal plants are widely used for the treatment of central nervous system disorders (Wheatley, 2005) but in some cases there is still lack of preclinical and clinical studies.

Central nervous system disorders have a great impact in society due to a general aging process of the population as well as lifestyle. Stress is one of the most prevalent psychological disorders in developed countries leading to other clinical features, such as anxiety, insomnia or depression.

Benzodiazepines (BZD) and selective serotonin reuptake inhibitors (SSRIs) are highly prescribed as anxiolytic and antidepressant drugs, respectively. BZD, such as diazepam, lorazepam or alprazolam produce calming effects via binding to GABA_A_ receptors, but they may also produce somnolence and cognitive impairment as adverse drug reactions. SSRIs (e.g., fluoxetin, paroxetin, citalopram) are prescribed as antidepressants because they are able to selectively block the serotonin transporter (SERT), but side effects include sexual dysfunction and neuropsychiatric disorders, such as suicide tendencies and sleep disorders. Both groups of medicines are also involved in withdrawal and “rebound effects” as a result of discontinuing their administration.

Certain EO are being used as anxiolytic remedies and the administration mode can be orally but also by inhalation or combined with massage. One of the most popular essential oils for mental disorders and anxiety is lavender *(Lavandula angustifolia* Miller or *Lavandula officinalis* Chaix). Lavender essential oil can be considered as one of the best-seller over the counter herbal remedies for anxiety, stress and depression. Studies reveal high content of linalool and linalyl acetate (Da Porto et al., 2009) and international organizations, such as the World Health Organization (WHO), the European Scientific Cooperative on Phytotherapy (ESCOP) or the European Medicines Agency (EMA) approve this medicinal plant to relieve stress, restlessness and anxiety (Community)^1^.

The growing awareness of the adverse effects of central nervous system drugs has led to develop new strategies and safer pharmacological agents in mental health. Enzymes, such as monoamine oxidase (MAO), proteins, such as the SERT and ligand-gated ion channels, such as GABA_A_ and NMDA receptors are therapeutic targets in neuropharmacology. With the aim to contribute to evidence-based herbal medicine we have studied the effects of lavender essential oil on pharmacological targets involved in anxiolytic and antidepressive properties as well as *in vitro* models of neurotoxicity.

## Methods

### Lavender essential oil (LEO) and chemicals

Pure lavender (*L. angustifolia*) essential oil was kindly supplied by Pranarom International (Belgium). Isolated monoterpenes (linalool, linalyl acetate) were purchased from Fluka. Enzymes, proteins and reagents were acquired from Sigma. Linalool and linalyl acetate were also tested when lavender essential oil had a clear and significant activity in the assays.

### Chemical profile by GC-MS

Although the essential oils are chemically characterized by Pranarom International, LEO was analyzed in the laboratory by GC-MS on an Agilent 6890N Network GC system coupled to a 5973 Network Mass Selective Detector, accelerating voltage -69.9 eV, recoding masses of 35.00–400.00. GC conditions: injector temperature: 150°C; temperature programme: start 50°C, 20°C/min to 300°C; column: HP5MS (5% phenylmethylsiloxane) capillary, 30.0 m × 250 μl × 0.25 μm nominal. Carrier gas: helium at 1.0 ml/min. A NIST library was used for comparison of MS data.

### Animals and brain membrane homogenates

Adult male Sprague Dawley rats were obtained from Taconic (Denmark). Ethical permission for the studies was granted by the Animal Welfare Committee, appointed by the Danish Ministry of Justice, and all animal procedures were carried out in compliance with the EC Directive 86/609/EEC and the Danish laws regulating experiments on animals. Rats were put down by competent staff, the heads were separated from the body and the brains were removed whereas cerebellum was discarded. The cortex were weighed and homogenized with an Ultra-Turrax using different buffers at 4°C and the tissue preparation in each case was carried out as earlier described (Ransom and Stec, 1988). The tissue homogenates were resuspended and stored in aliquots at −80°C until use.

### Bioassays regarding serotonergic targets

#### Monoamine oxidase-a inhibition (MAO-A Assay)

The bioassay was performed in a 96-well microplate (Saaby et al., 2009). Each well contained 50 μl of essential oil dilution or DMSO as blank (making a final concentration in the wells of 0.002, 0.01, 0.02, and 0.1%), 50 μl of chromogenic solution (0.8 mM vanillic acid, 417 mM 4-aminoantipyrine and 4 U/ml horseradish peroxidase in potassium phosphate buffer pH 7.6), 100 μl of 3 mM tyramine and 50 μl of 8 U/ml MAO-A. Absorbance was read at 490 nm every 5 min for 30 min. Background interferences were deducted in the same way described above but without MAO enzyme. Data was analyzed using GraphPad Prism. IC_50_ values could not be obtained because the higest tested concentration did not reach 50% of MAO inhibition. Clorgyline was used as positive control.

#### Serotonin transporter assay ([^3^H]-citalopram binding assay)

The assay was performed on the basis of the method from Nielsen et al. (2004). 25 μl of three different essential oil concentrations were mixed with 50 μl of 4 nM [^3^H]-Citalopram and 225 μl of rat cortex suspension making a final concentration of the essential oils in the assay of 0.08, 0.4, and 0.8%. All components were previously dissolved in buffer (50 mM Tris-base; 120 mM NaCl: 5 mM KCl at pH 7.5). Plastic tubes were placed on an ice bath, the reagents were added and the tubes were mixed and then left at room temperature (approximately 22°C) for 2 h. After incubation, 5 ml of ice cold buffer were added to the samples and they were filtered through GC-50 Advantec glass filters under vacuum and immediately washed once with additional 5 ml of ice cold buffer. Control test tubes (with buffer instead of EO) and blind tubes (with 25 μl of 120 μM paroxetine instead of EO) were done in every run in order to determine total and unspecific binding. The amount of radioactivity was determined transferring the glass filters into scintillation tubes and adding 4 ml of Ultimo Gold XR. The scintillation tubes were placed in the dark for 30 min before they were measured on a Tri-CARB 2100 TR analyzer. Three independent experiments were performed in triplicates. Non-radioactive citalopram was used as reference. Linalool and linalyl acetate were tested. The specific binding of [^3^H]-citalopram was determined using the formula: % binding = [dpm_(essential oil)_-dpm_(unspecific binding)_/dpm_(total binding)_-dpm_(unspeficic binding)_] × 100.

### Bioassays on ionotropic receptors

#### Affinity for GABA_A_ receptor ([^3^H]-Ro 15-1788 binding assay)

The membrane preparation was washed with ice-cold buffer (50 mM Tris-citrate pH 7.1). The suspension was centrifuged at 0–4°C for 10 min at 27,000 × *g*. The pellet was resuspended in Tris-citrate buffer (2 mg original tissue per ml) and used for the assay. 25 μl of ^3^H-Ro 15-1788 (flumazenil) was added to 25 μl of test solutions (10, 1, and 0.1 mg/ml) and 500 μl of membrane preparation. Total and unspecific binding was measured using buffer or diazepam (1 μM assay final concentration). After incubation for 40 min in an ice bath, 5 ml of ice-cold buffer was added to the samples and poured onto Adventic glass fiber filters (GC-50) under vacuum, and immediately washed with another 5 ml of ice-cold buffer. The amount of radioactivity of the filters was measured by conventional liquid scintillation counting using Ultimo Gold XR as scintillation fluid. Clonazepam was used as positive control.

#### Affinity for NMDA receptor ([^3^H]-CGP39653 binding assay)

Affinity for native NMDA receptors was determined using 2 nM [^3^H]-CGP 39653 (Sills et al., 1991) with some modification. On the day of the assay, frozen membranes were quickly thawed and homogenized in 30 volumes of ice-cold Tris-HCl buffer, pH 7.4 (50 mM containing 2.5 mM CaCl_2_), and centrifuged (48,000 × *g* for 10 min). This step was repeated three times. The final pellet was re-suspended in ice-cold buffer, corresponding to approximately 0.4–0.5 mg protein/ml. Binding was carried out in aliquots consisting of 25 μL [^3^H]-CGP 39653, 25 μL test solution, and 200 μL membrane suspension and incubated at 0° for 60 min. Non-specific binding was determined using 1 mM (*S*)-Glu. Binding was terminated by filtration through Whatman GF/C filters using a 96-well Packard Filter-Mate Cell Harvester and filters were washed with 3 × 250 μL of ice-cold buffer. After drying, 30 μL Microscint 0 (Perkin-Elmer) per well was added and the filter was counted on a Topcounter (Perkin-Elmer). Linalool and linalyl acetate were also tested in this assay.

### Neuroprotection on SH-SY5Y cells

#### SH-SY5Y neuroblastoma cell culture

Cells were cultured in DMEM (Gibco, ref. 41966-029) containing phenol red (PR), L-Glutamine (1 mM) and sodium pyruvate (1 mM) and was supplemented with 10% FBS (Gibco), penicillin (100 U/mL, Gibco) and streptomycin (100 U/mL, Gibco). Cells were maintained at 37°C in saturated humidity (5% CO_2_). Non-differentiated cells were plated on 48-well plates (Corning), at a density of 5 × 10^4^ cells per well and were used 48 h after seeding.

#### Aβ_25–35_solublespecies preparation

Aβ_25–35_fragment was purchased from Sigma-Aldrich laboratories (ref. A4559-1MG). 1 mg of Aβ_25–35_ fragment was dissolved in 1 mL of type II water and was frozen at -20°C until further use. Different concentrations of Aβ_25–35_ (5, 10 and 15 μM) were incubated in DMEM phenol red free (Gibco, 31053-028) at 37°C for 3 days to obtain the oligomeric forms.

#### Cell treatment and MTT cell viability assay

SH-SY5Y cells were treated with either different LEO concentration (0.05, 0.1, 0.5, and1 μL/mL) or incubation time (0, 2, and 24 h), followed by hydrogen peroxide (100, 200, 400, 800, and 1,600 μM), malonate (0 or 50 mM) or Aβ_25–35_(5, 10, and 15 μM).

Cell viability was examined by the 3,4,5-dimethylthiazol-2-yl-2,5-diphenyltetrazolium bromide (MTT) assay. MTT assay is a colorimetric assay for measuring the activity of cellular enzymes in living cells in response to potential toxic. After the cell treatment, culture medium was replaced by a solution of 5 mg/mL MTT (Sigma-Aldrich) in DMEM (Gibco, 31053-028). Cells were incubated with MTT solution for 2 h in the cell incubator (5% CO_2_ and 37°C). Then, the MTT solution was discarded and DMSO was added to the wells. Aliquots were transferred to a 96-well plate, and absorbance was measured at 595 nm in a plate reader. Results were expressed as percentages of non-treated control cells.

### Statistical analyses

Data are expressed as mean ± SE (figures) or as mean ±SD (tables) of at least three independent experiments performed in different days and in triplicates. GraphPad Prism was used to calculate IC_50_ values and to detect significant differences. Student *t*-test or ANOVA was performed for data analysis.

## Results

### Chemical profile by GC-MS

According to the GC-MS analyses, lavender essential oil mainly contained the following monoterpenes: linalyl acetate (52.1%), linalool (37.4%), geranyl acetate (5.4%), and β-caryophyllene (5.1%).

### Monoamine oxidase-a inhibition (MAO-A assay)

Inhibition of MAO A was not detected (data not shown).

### Serotonin transporter assay ([^3^H]-citalopram binding assay)

**Figure 1:**
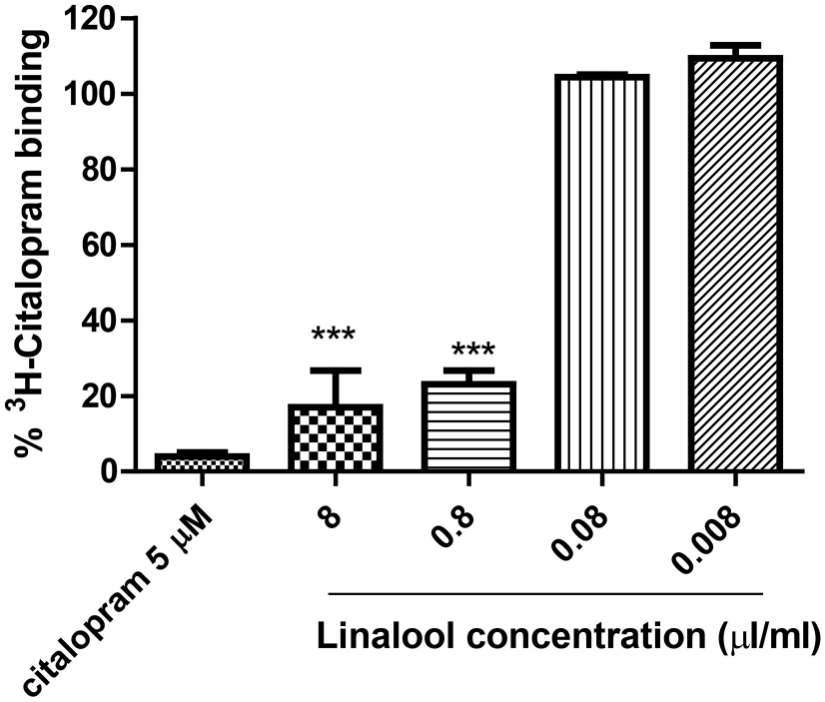
**Percentage of ^**3**^H-Citalopram binding induced by different concentrations of linalool on the SERT assay**. Linalyl acetate did not show affinity to the serotonin transporter (data not shown). ^***^p < 0.0001 vs. the lowest concentration tested.

The effects of LEO on the SERT are displayed in Table 1, showing moderate activity in this assay (citalopram IC_50_ value was 1.3 nM). LEO significantly displaced ^3^H-citalopram from binding to the SERT in a dose-dependent manner, which means that LEO might have an antidepressant-like effect via this specific transporter. This effect was also detected for linalool, one of the main constituents of lavender essential oil (Figure 1), but not for linalyl acetate.

**Table 1:** **Activity of lavender essential oil (LEO) on the serotonin transporter (SERT) and GABA_**A**_ ionotropic receptor**.

**Samples**	**SERT (%** ^**3**^**H-Citalopram binding)**		
	0.8 μl/ml	4 μl/ml	8 μl/ml
LEO	105.4 ± 0.9	77.6 ± 2.7^*^	37.8 ± 16.0^***^
	**GABA_A_ (% ^3^H-Flumazenil binding)**		
	0.045 μl/ml	0.45 μl/ml	4.5 μl/ml
LEO	100.3 ± 9.5	100.1 ± 0.8	105.2 ± 9.3

* p < 0.05;

*** *p < 0.001 vs. the lowest concentration tested (0.8 μl/ml in the SERT assay or 0.045 μl/ml in the GABA assay). Data are mean ± SD of three independent experiments performed in triplicates*.

### Affinity for GABA_A_ receptor ([^3^H]-Ro 15-1788 binding assay)

The affinity for the GABA_*A*_-benzodiazepine receptor, which may lead to a nerve calming effect, was not detected (Table 1). LEO had no effect in binding this ionotropic receptor. Clonazepam (IC_50_0.002 nM) was used as positive control drug.

### Affinity for NMDA receptor (CGP39653 binding assay)

**Figure 2:**
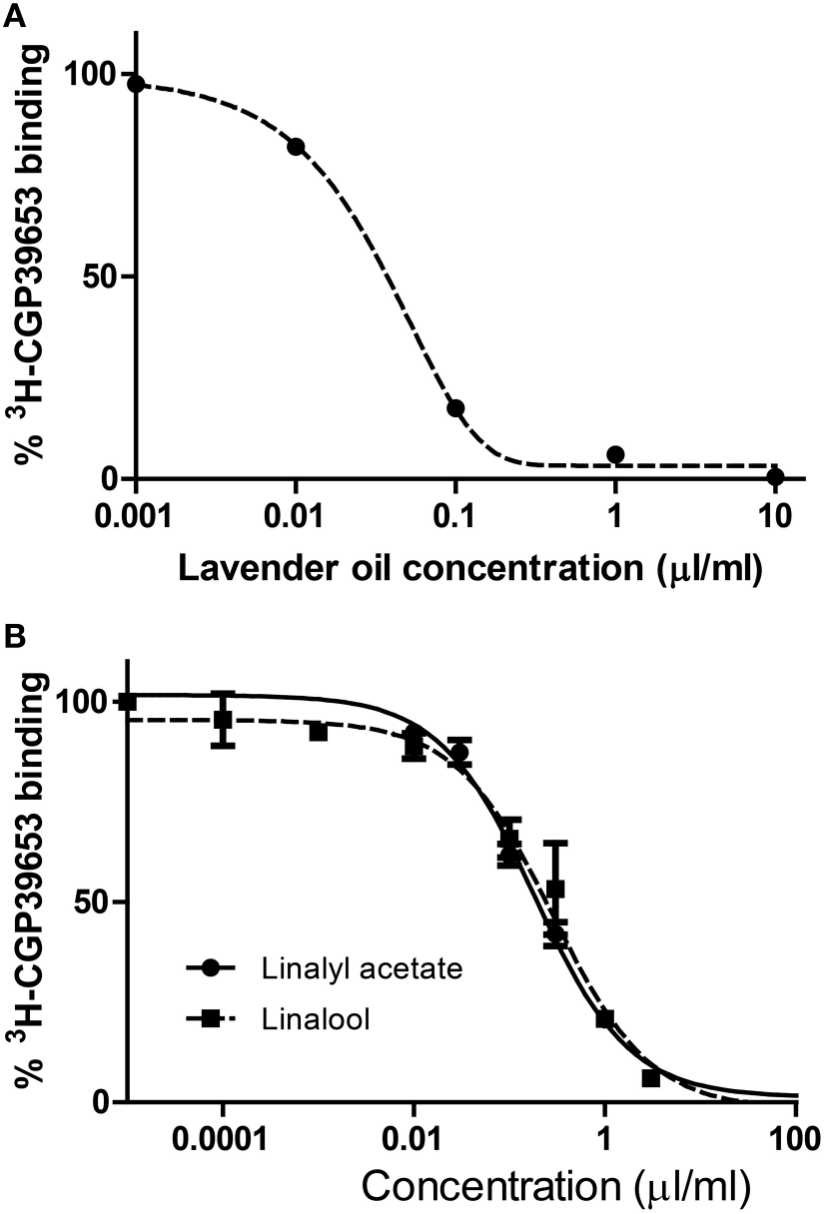
**Affinity of lavender essential oil (A)** and linalool and linalyl acetate **(B)** for the glutamate NMDA receptor. The displacement studies of [^3^H]CGP39653 were performed on membranes obtained from rat brain homogenates.

Lavender Essential Oil (LEO) was found to be active in the CGP39653 binding assay. Figure 2 shows the profile of the affinities for the NMDA receptor and Table 2 presents results in terms of IC_50_ and K_i_ values. It can be observed that lavender was significantly active showing a clear dose-response activity (Figure 2A). In this case, linalool and linalyl acetate were also tested, showing binding properties to the NMDA receptor (Figure 2B).

**Table 2:** **IC_**50**_ and K_***i***_ values of lavender essential oil (LEO) and monoterpenes in the [^**3**^H]CGP39653 binding assay**.

**Essential oil**	**IC_50_ (μl/ml)**	**K_*i*_ (μl/ml)**
LEO	0.04 ± 0.09	0.026 [0.022;0.030]
Monoterpenes	IC_50_ (mM)	K_i_ (mM)
Linalyl acetate	0.74 ± 0.18^**^	0.54 [0.47;0.62]
Linalool	2.97 ± 0.63	2.3 [2.1; 2.6]

** *p < 0.01 vs. linalool; each IC_50_ value, determined from the “dose-response” curve, has been converted into the K_i_ value using the Cheng Prusoff equation: (K_i_ = IC_50_ × [1/([L]/K_D_) + 1)]. (S)-Glu has a Ki value of 0.02 μM*.

### Effects of lavender oil against hydrogen peroxide in SH-SY5Y cells

**Figure 3:**
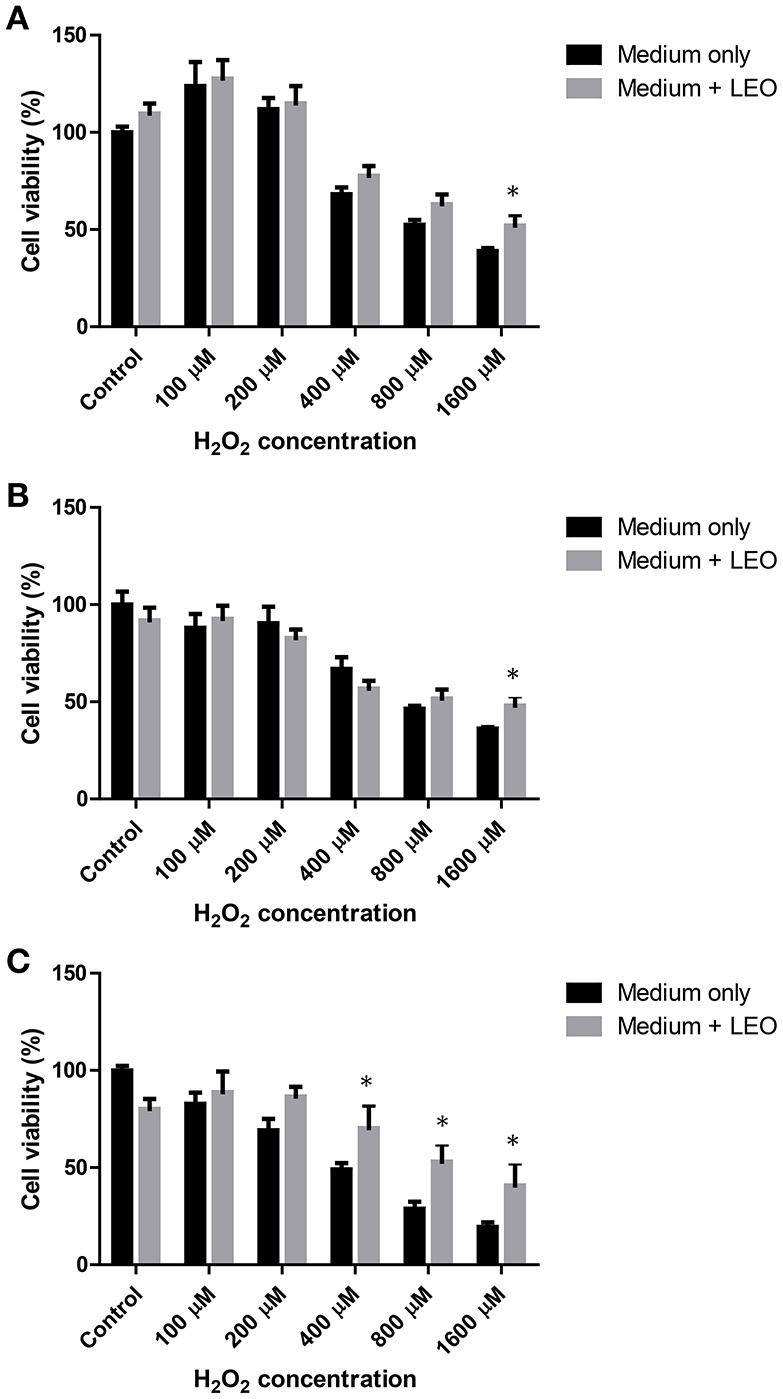
**Effect of 1 μl/ml lavender essential oil (LEO) on hydrogen peroxide induced toxicity on SH-SY5Y cells at 0 (A)**, 2 **(B)**, and 24 h **(C)**. ^*^p < 0.05 vs. medium only.

As depicted in Figure 3A, when 1 μl/ml lavender oil was incubated 0 h before the addition of hydrogen peroxide (H_2_O_2_), lavender oil only produced a significance difference (^*^p < 0.05, Two-way ANOVA) at 1,600 μM of H_2_O_2_. Same result was obtained when lavender oil was incubated 2 h prior the addition of H_2_O_2_ (^*^p < 0.05, Two-way ANOVA) (Figure 3B). As shown in Figure 3C, when lavender oil was incubated 24 h prior the addition of H_2_O_2_ a significant reversion of cell death was obtained at 400, 800, and 1,600 μM of H_2_O_2_ (^*^p < 0.05, Two-way ANOVA).

### Effects of lavender oil against malonate in SH-SY5Y cells

**Figure 4:**
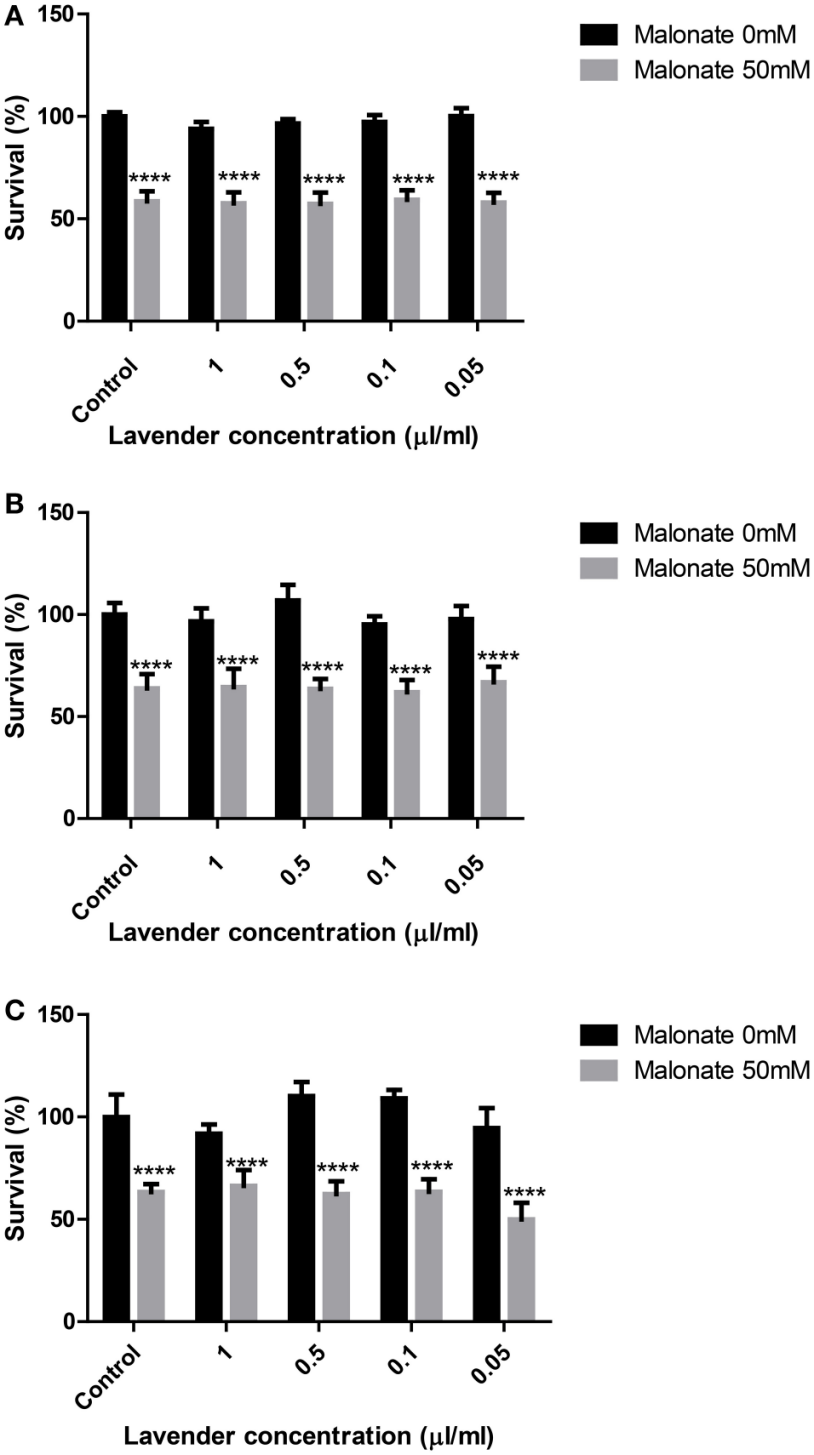
**Effect of lavender essential oil on malonate induced toxicity on SH-SY5Y cells at 0 (A)**, 2 **(B)**, and 24 h **(C)**. ^****^p < 0.0001 vs. 0 mM malonate in the respective lavender concentration.

Lavender oil did not protect from malonate induced toxicity in any of the concentrations (0.05, 0.1, 0.5, and 1 μL/mL) or incubation times tested (0, 2, or 24 h; Figure 4).

### Effects of lavender oil against Aβ_25–35_ in SH-SY5Y cells

**Figure 5:**
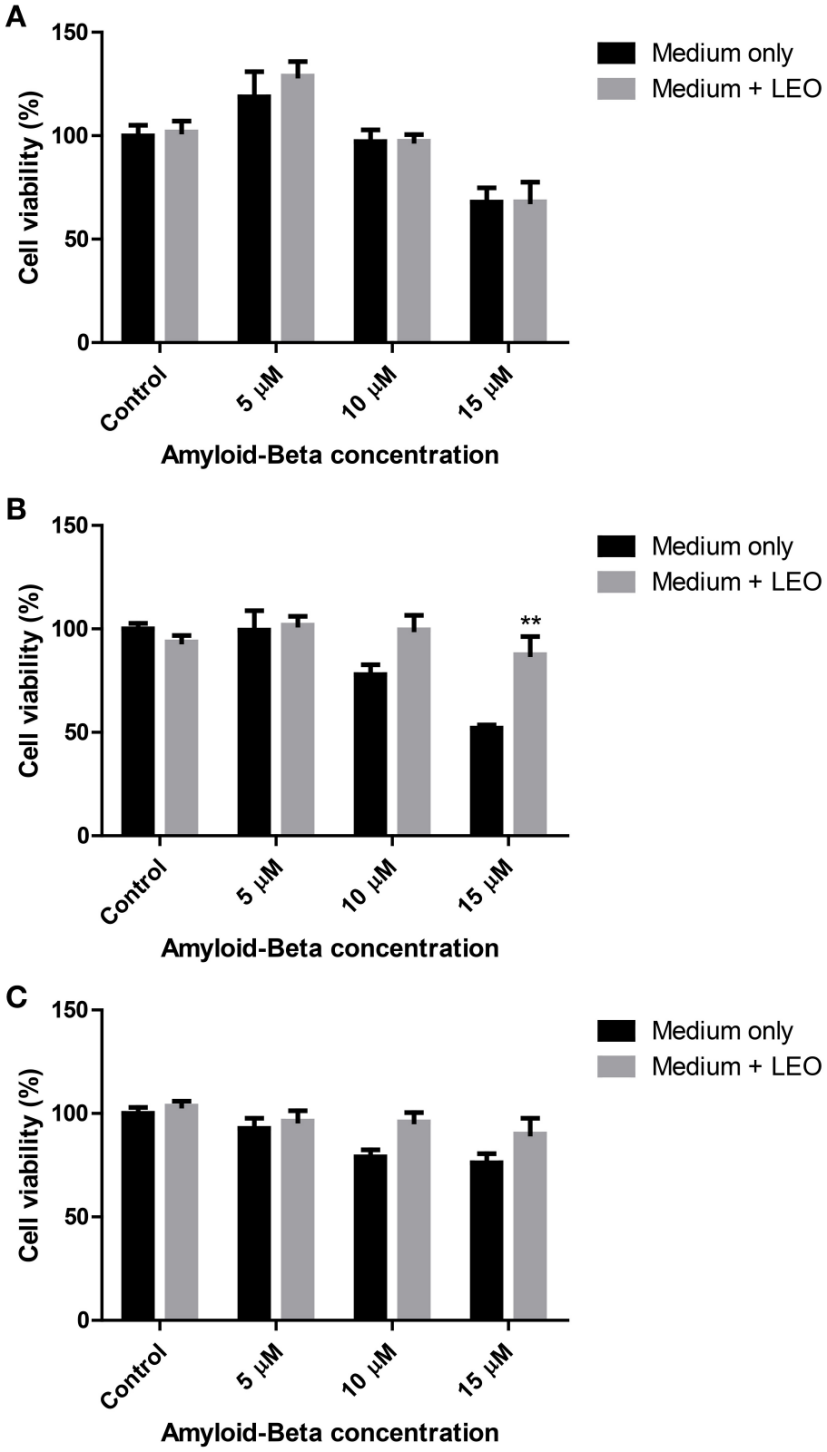
**Effect of 1 μl/ml lavender essential oil (LEO) on beta-amyloid induced toxicity on SH-SY5Y cells at 0 (A)**, 2 **(B)**, and 24 h **(C)**. ^**^p < 0.01 vs. 15 μM beta-amyloid (medium only).

As observed in Figure 5, lavender oil only produced a significant effect against Aβ_25–35_15 μM induced toxicity when it was incubated 2 h before toxin addition (^**^p < 0.01, Two-way ANOVA; Figure 5B).

## Discussion

Lavender is used in pharmacy, phytotherapy and aromatherapy to treat central nervous system disorders, such as anxiety, stress, and sleep disorders. This essential oil is one of the best-selling natural remedies and a common therapeutic tool for physiotherapists and chiropractors.

There has recently been an increase in the use of natural therapies due to well-known side effects of common CNS drugs, such as (BZD) and selective serotonin re-uptake inhibitors (SSRI). Although BZD and SSRI have been widely used in neuropharmacology, there is a need of developing new therapeutic tools that play a role both in prevention and treatment of mild CNS disorders.

In this sense, aromatherapy and some essentials oils have been used for decades to induce nerve-calming effects and mood-enhancing properties (Dobetsberger and Buchbauer, 2011). Aromatherapy is a form of complementary and alternative medicine (CAM) that uses plant essentials oils to affect the mood or health of the patient (Wheatley, 2005) and some clinical studies suggest that inhaled or topically applied essential oils like lavender exert psychological and central nervous system effects (Perry and Perry, 2006). It has also recently been stated that essential oils may behave as antidepressants due to playing an important role on neurotransmitter pathways, mainly in the serotonin system (Lv et al., 2013).

Certain studies have reported pharmacological properties of essential oils but there is little research on the molecular mechanisms underlying the CNS effects. The ability of lavender essential oil to interact with neuropharmacological targets, such as MAO-A, the SERT and ionotropic receptors (GABA_A_ and NMDA) has been tested, as well as the protective potential against neurotoxic agents, such as hydrogen peroxide, malonate and amyloid peptide.

It was surprising that LEO had the capacity to bind to the NMDA receptor, which was measured through the CGP39653 binding assay. CGP39653 is a competitive antagonist, being currently the ligand of choice for labeling NMDA receptors. NMDA receptors are neurochemically classified as ionotropic glutamate receptors (iGLURs) and are involved in certain neurological and psychiatric disorders, such as epilepsy, sustained-seizure damage, Parkinsonism, etc. For this reason iGLURs are considered pharmacological targets in drug research and development. LEO was able to displace CGP39653 binding in a dose-dependent mode, which means that this oil may exert nerve-calming effects via modulating NMDA receptors. This is the first time that this affinity is reported and this fact could explain the anti-agitation properties that have been found for these products in animal and some clinical studies (Bradley et al., 2009; Faturi et al., 2010; Kasper et al., 2010; Tsang and Ho, 2010; Woelk and Schlaefke, 2010; Chioca et al., 2011; Goes et al., 2012; Hritcu et al., 2012; Schuwald et al., 2013). NMDA-receptor activation by glutamate is also involved in neurotoxicity so our essential oil might exert neuroprotection through the blockade of this ionotropic receptor.

According to our data, these results are in the range of other natural products that have been reported to show activity in this target (Cho et al., 2001; Pedersen et al., 2008; Marchetti et al., 2011).

Lavender essential oil has shown activity in animal and human studies but according to Koulivand et al. (2013), due to methodological inadequacies, small sample sizes, variations on administration methods or the absence of placebos or control groups in the studies, more standard experiments are needed to confirm the benefits of lavender in CNS disorders. The molecular mechanisms involved in lavender effects have been suggested in other studies. For example, Huang et al. (2009) suggest that lavender essential oil reversibly inhibited GABA-induced currents in a concentration-dependent manner (0.01–1 mg/mL), whereas no inhibition of NMDA- or AMPA-induced currents was noted. In a recent study, Schuwald et al. (2013) identified a standarized lavender essential oil (Silexan) as a potent anxiolytic inhibiting voltage dependent calcium channels in synaptosomes, primary hippocampal neurons and stably overexpressing cell lines, in the same range as pregabaline.

Lavender essential oil is known to contain monoterpenes like linalool and linalyl acetate, which seem to be responsible for the activity according to our data and previous works (Cline et al., 2008; Linck et al., 2010; Souto-Maior et al., 2011). The capacity of linalool to interact with the glutamatergic system and the NMDA receptor is not new as it has been previously described by other authors (Elisabetsky et al., 1995, 1999; Silva Brum et al., 2001; Aprotosoaie et al., 2014); however, the ability of linalyl acetate to bind the NMDA receptor has not been found to be reported in previous works. It seems that lavender essential oil anxiolytic effects is due to the fact that its main monoterpenes, linalool and linalyl acetate, interact with the NMDA receptor.

It is very difficult to establish structure activity relationships because linalyl acetate was inactive in the SERT assay, whereas the activity of this compound was slightly higher than linalool in the NMDA binding receptor assay. Linalool and linalyl acetate are secondary metabolites classified as monoterpenes with a similar structure consisting of a linear hydrocarbon chain of 10 carbons (linalool) or 11 (linalyl acetate), being linalyl acetate the ester of linalool. The free hydroxyl group in linalool seems to be determinant for the activity in the SERT. On the contrary, the activity on the NMDA receptor is increased when the acetate group exists in the compound named linalyl acetate; limonene, another monoterpenic compound found at high concentration in *Citrus* essential oils, has also shown anxiolytic-like activity with other targets involved (de Almeida et al., 2012; Lima et al., 2013).

Other essential oils have been demonstrated to produce changes in brain neurotransmitters which could explain its anti-agitation properties. For example, ylang-ylang (*Cananga odorata*) essential oil has shown anxiolytic effects in male mice decreasing the dopamine concentration in the striatum and increasing the serotonin concentration in the hippocampus (Zhang et al., 2016). Surprisingly, linalool is one of the main components of this essential oil. The essential oils obtained from *Eugenia uniflora* has also demonstrated antidepressant-like activities in animal models with the serotonergic and adrenergic systems being involved (Victoria et al., 2013). *Melissa officinalis* essential oil, which is also used as a natural sedative, has demonstrated a depressant effect on neurotransmission but no inhibition of NMDA- or AMPA-induced currents was detected (Abuhamdah et al., 2008). *Citrus aurantifolia* (bitter orange) essential oil has also exhibited anxiolytic-like effects mediated by 5-HT(_1A_)-receptors (Costa et al., 2013). All these works put forward the idea that the serotonergic system is involved in the nerve calming effects of essential oils.

We have also observed that lavender and linalool inhibits serotonergic targets, such as the SERT, which might explain why lavender has shown antidepressant-like effects in animal and human models (Cavanagh and Wilkinson, 2002; Akhondzadesh et al., 2003; Hritcu et al., 2012). However, we did not observe effects on the GABA_A_-receptor, which is in accordance with results from a recent study (Chioca et al., 2013).

This study has been completed using neuroblasts from neural human tissue (SH-SY5Y) exposed to different neurotoxic agents. Lavender was able to protect the cells from the toxic insult generated by hydrogen peroxide although it was not capable to reduce malonate toxicity. Results from amyloid-β peptide induced toxicity are not conclusive. However, this activity seems to be in accordance with a previous study where lavender essential oil showed neuroprotective properties against hydrogen peroxide induced toxicity in PC12 cells (Xu et al., 2016). The authors conclude that lavender protected the cells reducing LDH, NO release, intracellular ROS accumulation and MMP loss.

## Conclusion

Our study reveals for the first time that lavender exerts receptor binding affinities with a relevant activity on the NMDA receptor. According to our data, we can state that the anti-agitation and antidepressant activities of lavender may be attributed at least in part to the NMDA receptor modulation as well as an inhibition of the SERT. Lavender essential oil also protected SH-SY5Y cells from hydrogen peroxide induced neurotoxicity.

## Author contributions

VL conceived the study, performed *in vitro* pharmacological activities (enzyme inhibition tests, affinities to receptors and transporters), carried out data analysis and wrote the manuscript. BN run the NMDA activity assay. MS and MR perfomed the cell assays neuroblasts. AJ supervised all work.

### Conflict of interest statement

The authors declare that the research was conducted in the absence of any commercial or financial relationships that could be construed as a potential conflict of interest.

## Footnotes

^1^Community herbal monograph on Lavandula angustifolia Mill., aetheroleum., EMA/HMPC/143181/2010 (http://www.ema.europa.eu/ema/).

## Abbreviations

CNS: central nervous system
LEO: lavender essential oil
GABA: γ-aminobutyric acid
iGLURs: ionotropic glutamate receptors
MAO-A: monoamine oxidase A
NMDA: n-methyl-D-aspartate
SERT: serotonin transporter
SSRI: selective serotonin reuptake inhibitor.
